# Choosing the right path: enhancement of biologically relevant sets of genes or proteins using pathway structure

**DOI:** 10.1186/gb-2009-10-4-r44

**Published:** 2009-04-24

**Authors:** Reuben Thomas, Julia M Gohlke, Geffrey F Stopper, Frederick M Parham, Christopher J Portier

**Affiliations:** 1 Environmental Systems Biology Group, Laboratory of Molecular Toxicology, National Institute of Environmental Health Sciences, RTP, NC 27709, USA; 2 Department of Biology, Sacred Heart University, Fairfield, CT 06825, USA

## Abstract

A method is proposed that finds enriched pathways relevant to a studied condition using the measured molecular data and also the structural information of the pathway viewed as a network of nodes and edges. Tests are performed using simulated data and genomic data sets and the method is compared to two existing approaches. The analysis provided demonstrates the method proposed is very competitive with the current approaches and also provides biologically relevant results.

## Abstract 2

A method is proposed that finds enriched pathways relevant to a studied condition, using molecular and network data.

## Background

Data on the molecular scale obtained under different sampling conditions are becoming increasingly available from platforms like DNA microarrays. Generally, the reason for obtaining molecular data is to use these data to understand the behavior of a system under insult or during perturbations such as occurs following exposure to certain toxicants or when studying the cause and progression of certain diseases. Toxins or diseases will hereafter be commonly referred to as perturbations to the biological system. Genomics is capable of providing information on the gene expression levels for an entire cellular system. When faced with such large amounts of molecular data, there are two options available that can enable one to focus on a small number of interesting sets of genes or proteins. One can cluster the data [1] and use the clusters to identify sets of genes that were significantly affected by the perturbations. This represents an unsupervised approach. Other similar approaches include principal component analysis [2] and self-organizing maps [3].

Alternatively, biologically relevant sets of genes/proteins are deduced to exist *a priori *in the form of biochemical pathways and cytogenetic sets. A supervised approach can be linked with the data to identify these *a priori*-defined sets that are significantly affected by the perturbations seen in the data. The method proposed in this paper is an example of this approach applied to the scenario of distinguishing between two conditions (such as normal patient versus disease patient, or unexposed versus exposed). The data we wish to link to a given set of pathways are assumed to be genomic data such as gene expression levels or the presence of gene polymorphisms known to be associated with diseases.

Supervised approaches for the identification of biologically relevant gene expression sets have typically been identified as 'gene set' or 'pathway enrichment' methods in the literature. Recent years have seen significant work done on proposals for new approaches guided by criticisms and limitations of the existing ones; references [4-8] provide a critical review of the existing methods in terms of their different features, such as the null hypotheses of the underlying statistical tests used and the independence assumption between genes. These reviews essentially inform us that the pathway enrichment methods can be viewed as falling on two sides of a number of different coins. A few of these classifications are given below.

Firstly, methods could be interested in testing either whether the genes in a specific pathway of interest are affected as a result of a treatment (the implied null hypothesis has been referred to as 'self-contained' [4] or denoted as 'Q2' [9]) or whether the genes in the pathway of interest are more affected than the other genes in the system (this implied null hypothesis has been referred to as 'competitive' [4] or as 'class 1, 2, 3' [6] or denoted as 'Q1' [9]). There are of course good reasons for preferring either of these null hypotheses. One would prefer the 'competitive' hypothesis if the treatment had a wide ranging impact on the genes in the system. This could have an undesirable consequence of having randomly chosen (and hence not biologically relevant) sets of genes attaining significance for the 'self-contained' tests; a nice illustration of a case like this is provided in [10]. One could use a 'self-contained' test if the belief is that the treatment had quite a restricted impact on the genes in the system and/or if their only focus is on one or a small number of pathways.

Some of the pathway enrichment methods treat the genes in the system as being independent of each other [7,9,11-22]. Ignoring the gene-gene correlations has been shown to have the effect of elevated false-positive discoveries [4,6]. However, the need to prioritize the different biological pathways with respect to their relevance to the treatment and the lack of a sufficient number of biological replicates (one in some cases) may force the need for this independence assumption. Examples of methods that try to take into account the gene-gene correlations include [6,9,10,23-37].

Pathway enrichment methods can be distinguished by the use or the absence of an explicit gene-wise statistic to measure the gene's association with the treatment in determining a pathway's relevance to the treatment. Examples of gene-wise statistics used include the two-sample *t*-statistic, log of fold change [35], the significance analysis of microarrays (SAM) statistic [25] and the *maxmean *statistic [10]. Methods like those in [24,30,31,34,37,38] treat the problem as a multivariate statistical one and avoid the need for an explicit definition of a gene-wise statistic.

The method proposed in this paper defines versions for both the 'self-contained' and the 'competitive' null hypotheses and utilizes the idea of the *maxmean *statistic [10]. It improves upon the previous methods by its use of structural information present in biochemical pathways. A pathway is said to have structural information if its components can be placed on a network of nodes and edges. For example, a gene set corresponding to a pathway can be viewed to be associated with a network where the nodes represent the gene products (that is, proteins, protein complexes, mRNAs) while the edges represent either signal transfer between the gene products in signaling pathways or the activity of a catalyst between two metabolites in metabolic pathways.

Classic signal transduction pathways, such as the mitogen-activated protein kinase (MAPK) pathways, transduce a large variety of external signals, leading to a wide range of cellular responses, including growth, differentiation, inflammation and apoptosis. In part, the specificity of these pathways is thought to be regulated at the ligand/receptor level (for example, different cells express different receptors and/or ligands). Furthermore, the ultimate response is dictated by the downstream activation of transcription factors. Alternatively, intermediate kinase components are shared by numerous pathways and, in general, do not convey specificity nor do they directly dictate the ultimate response (see [39] for a review). Therefore, we test the value of implementing a Heavy Ends Rule (*HER*) in which the initial and final components of a signaling pathway are given a higher weight than intermediate components.

Signal transduction relies on the sequential activation of components in order to implement an ultimate response. Therefore, we hypothesize that activation of components that are directly connected to each other in a pathway conveys greater significance than activation of components that are not closely connected to each other. Therefore, we also test the implementation of a Distance Rule (*DR*) scoring rule in which genes that are closely connected to each other are given a higher score.

The use of structural information based on an underlying network in an analysis of gene expression data is not new. Similar ideas have been used to identify activated pathways from time profile data (here the attempt was to distinguish between two phenotypes) [40], while structural information of the pathways has been used to enhance the clusters deduced from the gene expression data [41] and to find differentially expressed genes [42]. The study by Draghici *et al*. [43] appears to be the only existing work that incorporates pathway network information to the problem of pathway enrichment. However, this appears to be limited by the need to define an arbitrary cut-off for differential expression, the assumption of independence between genes and the parametric assumption of an exponential distribution for computing the significance.

## Results and discussion

The method proposed in this paper is named 'structurally enhanced pathway enrichment analysis' (*SEPEA*). It is a pathway enrichment method that incorporates the associated network information of the biochemical pathway using two rules, the *HER *and *DR*. *SEPEA *provides three options for null hypothesis testing (*SEPEA_NT1*, *SEPEA_NT2 *and *SEPEA_NT3*) that depend on the goal of the pathway enrichment analysis and the properties of genomic data available. *SEPEA_NT1 *and *SEPEA_NT2 *require multiple array samples per gene and are tests that take into account inherent gene-gene correlations. *SEPEA_NT3 *just requires a summary statistic per gene (that indicates association with the treatment) but assumes that genes are independent of each other. The need for the test *SEPEA_NT3 *is motivated by the fact that there are situations where the data are just not sufficient to estimate gene-gene correlations, such as the case where the only information available is whether a gene is or is not affected by the treatment; analyzing the situation of having a set of gene polymorphisms known to be associated with breast cancer is one such example. *SEPEA_NT1 *and *SEPEA_NT3 *are proposed to be used in situations where the goal is to compare the genes in the pathway of interest to the other genes in the system in terms of their associations with the treatment. *SEPEA_NT2 *is used for analyses involving only the genes in the pathway in relation to the treatment. The main objective of this paper is to demonstrate the utility of incorporating pathway network information in a pathway enrichment analysis. Therefore, comparisons are made with results from corresponding versions of *SEPEA *that do not use the network information - SEPEA_NT1*, SEPEA_NT2* and SEPEA_NT3*. In addition, two literature methods are used for comparison with the results from *SEPEA_NT1 *- gene set enrichment analysis (*GSEA*) [35] and the *maxmean *method [10] - the null hypotheses of *GSEA *and *maxmean *being very similar to *SEPEA_NT1*.

### Motivation for the Heavy Ends Rule score

**Figure 1:**
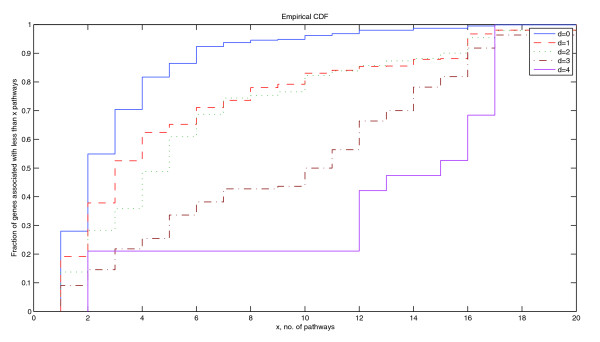
Empirical distribution function of number of pathways associated with genes at given distances from terminal nodes. Empirical cumulative distribution function of the number of pathways that are associated with genes that have gene products located at a given distance, *d *(= 0, 1, 2, 3, 4), from a terminal node of the pathway network. Gene products that are at a distance *d *= 0 are the terminal gene products. The data used were those of all the genes associated with human signaling pathways in the KEGG pathway database [44].

By giving greater weight to genes whose products are nearest to the terminal gene products of a pathway, the *HER *score gives more weight to genes specific to a particular pathway. This is illustrated in Figure 1, which uses the concept of terminal gene products. They are gene products like either receptors that initiate the pathway activity or transcription factors that are made to initiate transcription as a result of the pathway activity (see Materials and methods for a more mathematical definition). The genes involved in each of the signaling pathways in the Kyoto Encyclopedia of Genes and Genomes (KEGG) pathway database [44] were evaluated for the position of their gene products with respect to the terminal gene products and the total number of signaling pathways that these genes are involved in. It is clear from Figure 1 that genes associated with products that are closer to the terminal gene products are more pathway-specific.

### Justification for the Distance Rule score

To illustrate the utility of the *DR *as a scoring method, we consider the linkage between the full set of pathways in KEGG [44]; that is, the pathways themselves can be viewed to be part of a higher level network, the nodes of which are pathways while the edges indicate the transfer of signal or material between pathways (Figure S1 in Additional data file 2). For example, the MAPK signaling pathway and the p53 signaling pathway can be considered to be linked. It seems reasonable to expect that after perturbation of the system, the affected pathways that are linked are more likely to respond similarly. We test this intuition using different microarray data (from the Gene Expression Omnibus (GEO) database [45] in a statistical test on the above network of pathways. The details are provided in the Materials and methods section. The *P*-values for the eight comparisons (estimated using 1,000 random networks) are given in Table 1. Significant *P*-values across the comparisons support our use of the *DR *as a reasonable score for differentiating between pathways.

**Table 1:** Significance of observed pattern of *DR *scores across all KEGG pathways for different GEO datasets

GEO accession number	Description	*P*-value
[GEO:GDS2744]	MCF-7 breast cancer cells - dioxin treatment versus control	0.005
[GEO:GDS2649](1)	Early HIV infection CD8+T cells versus uninfected	<0.001
[GEO:GDS2649](2)	Chronic HIV infection CD8+T cells versus uninfected	0.001
[GEO:GDS2649](3)	Non-progressive HIV infection CD8+T cells versus uninfected	0.004
[GEO:GDS2852](1)	Bronchial A549 cells - cytokine treatment at 0 h versus control	0.001
[GEO:GDS2852](2)	Bronchial A549 cells - cytokine treatment at 4 h versus control	<0.001
[GEO:GDS2852](3)	Bronchial A549 cells - cytokine treatment at 12 h versus control	<0.001
[GEO:GDS2852](4)	Bronchial A549 cells - cytokine treatment at 24 h versus control	0.016

Different control versus treated conditions in three microarray datasets indicated by the GDS accession numbers [GEO:GDS2744], [GEO:GDS2649] and [GEO:GDS2852] from the GEO database were used [45] to compare the *DR *scores across all the pathways on the pathway network (Figure S1 in Additional data file 2) using the *meta_DR *term in Equation 9. The *P*-value for the significance of *meta_DR *is computed using 1,000 random networks whose generation is described in the Materials and methods section.

### Analysis using simulated data

Simulated data were generated from two pathway networks having different patterns of correlation between the various genes in the pathway, with each network having genes in a pool of genes representing a biological system. The pair of networks and the correlation patterns of genes in the pathway, denoted by pattern numbers, are listed in Table 2. Patterns 1, 2, 3 and 4 have non-zero correlation between a subset of genes in the system. All genes in pattern 5 are assumed to be independent of each other. Patterns 1 and 3 are biased to the scoring rules proposed here whereas patterns 2 and 4 are not. The treatments had the effect of increasing (as given in the variable, *pert*) the expressions of certain genes in the system.

**Table 2:** Simulation conditions for comparing various methods for pathway enrichment

Pattern number	Network	Correlated set (Σ)	Target set (Φ)
1	*Linear*	{*g*_1_,..., *g*_9_}	
2	*Linear*	*U*^*L*^	
3	*ErbbSignaling*		
4	*ErbbSignaling*	*U*^*E*^	
5	*Linear*	Ø	

Different correlation patterns (1-5) considered for the generation of simulated data along with the underlying networks, the set of correlated genes, Σ, and the set of genes that are the targets of the treatment, Φ. *U*^*L *^denotes a uniformly randomly drawn set of nine genes drawn from the set of genes associated with the pathway displayed in Figure 1a. *V*_*41*_^*L *^denotes a set of 41 randomly drawn genes from the set of 470 genes not associated with the pathway displayed in Figure 1a. *U*^*E *^denotes a uniformly randomly drawn set of seven genes drawn from the set of genes associated with the pathway displayed in Figure 1b. *V*_*3*_^*E *^ denotes a set of three randomly drawn genes from the set of 413 genes not associated with the pathway displayed in Figure 1b. Ø denotes the empty set. The symbol ∪ denotes the set union operation.

Table 3 gives estimates of the type 1 errors of the five methods, at the 0.01 and 0.05 significance levels, for patterns 1 and 5. Table 4 gives estimates of the power of the *SEPEA_NT1*, *GSEA *and *SEPEA_NT2 *methods at 0.01 and 0.05 significance levels, for a *pert *value of 1.2 and for patterns 1-4. The empirical sizes of the methods *maxmean *and *SEPEA_NT3 *do not match their nominal sizes. So the results are provided at empirical sizes of 0.07 and 0.05 (corresponding to a nominal size of 0.001 for both cases).

**Table 3:** Type 1 error of different pathway enrichment methods

Pattern number	*A*	*SEPEA_NT1*	*GSEA*	*Maxmean*	*SEPEA_NT2*	*SEPEA_NT3*
1	0.01	8	5	85	13	135
	0.05	36	51	187	44	266
5	0.01	7	12	12	7	15
	0.05	51	45	48	52	53

Type 1 errors (in terms of the number of experiments out of 1,000 that gave *P*-values for the randomization tests below *α *= 0.01 and 0.05 levels) for each of the five methods and for correlation patterns 1 and 5.

**Table 4:** Power of different pathway enrichment methods

Pattern number	*A*	*SEPEA_NT1*	*GSEA*	*Maxmean*	*SEPEA_NT2*	*SEPEA_NT3*
1	0.01 0.05	328 610	188 510	52	357 686	321
2	0.01 0.05	271 505	189 508	37	295 580	39
3	0.01 0.05	344 692	222 496	32	347 712	480
4	0.01 0.05	166 361	212 468	32	157 379	11

Power estimates for the *SEPEA_NT1*, *GSEA *and *SEPEA_NT2 *methods (in terms of the number of experiments out of 1,000 that gave *P*-values for the randomization tests below nominal sizes of *α *= 0.01 and 0.05). The estimates for *maxmean *are given at an empirical size of 0.07 (nominal size of 0.001) and those for *SEPEA_NT3 *at an empirical size of 0.05 (nominal size of 0.001). These are results from simulations in which the treatment resulted in an over-expression of the mean expression of the target genes by the factor *pert *= 1.2. The methods were evaluated on correlation patterns 1-4.

Only patterns 1 and 5 were used to analyze the type 1 error behavior because they represented the two scenarios (presence or absence of gene-gene correlations) where pathway enrichment methods have been shown to have different behaviors [4,10]. Because of the presence of correlations in the data, *SEPEA_NT3 *gives an incorrect type 1 error value for pattern 1 (Table 3). As has been stated previously, in spite of this incorrect behavior, there are situations (like those in which the only information available for each gene is a summary statistic representing the effect of the treatment) where methods like *SEPEA_NT3 *need to be used in order to create relevant hypotheses regarding affected processes due to the treatment. *SEPEA_NT1*, *SEPEA_NT2 *and *GSEA *do maintain the right type 1 error behavior in both the presence and absence of gene-gene correlations. In the presence of gene-gene correlations, the *maxmean *method [10] also does not maintain the appropriate type 1 error behavior. As expected, the power estimates of all three *SEPEA *methods for patterns 1 and 3 were significantly higher (*P *< 0.05, two-sample test of proportions) than those for patterns 2 and 4, respectively. The power estimates for patterns 1 and 3 using *SEPEA_NT1 *were higher than those for *GSEA*, demonstrating improvement in the ability to detect these biologically relevant patterns. For the other two 'not-so-relevant' patterns (2 and 4), *SEPEA_NT1 *was not always more powerful than the *GSEA *method. This loss of power can again be explained by the bias of *SEPEA *to detect conditions favored by the scoring rules. For example, the power estimates of *SEPEA_NT1 *were also higher than those for *GSEA *[35] for pattern 2 whereas this was not the case for pattern 4. At an empirical size of 0.07, *maxmean *does not appear to be competitive with the other methods. *SEPEA_NT1 *also provides a more powerful method than *GSEA *on pattern 1 across a range of perturbation levels and signal to noise levels (Tables S3 and S4 in Additional data file 1). In addition, power results for four other correlation patterns are presented in Table S2 in Additional data file 1.

### Analysis using lung cancer data

**Figure 2:**
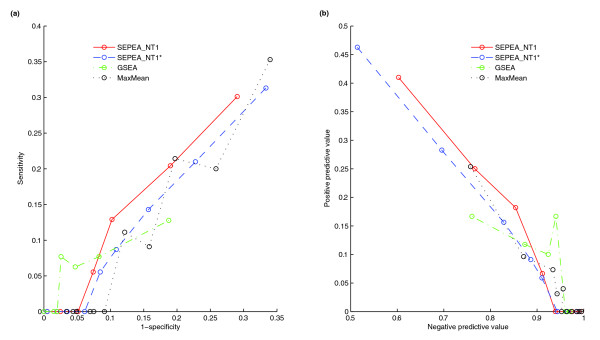
Receiver-operator characteristic and positive predictive power versus negative predictive power plots for lung cancer data. **(a) **Sensitivity versus '1 - specificity' of enriched pathways that are predictive of survival from lung cancer for four methods: *SEPEA_NT1*, SEPEA_NT1*, *GSEA *and *maxmean*. SEPEA_NT1* is the same analysis as *SEPEA_NT1 *except that the pathway network information was not used. **(b) **Positive predictive power (ppp) versus negative predictive power (npp) for the same data and using the same methods of analysis as in (a).

The study by Raponi *et al*. [46] analyzes gene expression data taken from 130 lung cancer patients in different stages of the disease. They also provide survival times for each patient. The data are divided into two groups of 85 patients (training set) and 45 patients (test set). This was done such that the proportion of patients in each stage was approximately the same for the two groups. Using these data, the Cox proportional hazards statistic is computed for each gene on the microarray (indicating how predictive it is of the survival time of a patient). The next logical step is then an attempt to find what biochemical pathways are predictive of survival. All of the human KEGG [44] pathways are used in this analysis. The methods used were *SEPEA_NT1*, *GSEA *and *maxmean*. Also, to estimate the value of including information on the network structure, *SEPEA_NT1 *was applied to the data assuming that all the genes in the pathway are given equal weight and the *DR *score is zero. This analysis is denoted by SEPEA_NT1*. The goal of our analysis is to evaluate consistency in choosing 'significant' pathways found using the training set versus the test set. Curves for sensitivity versus '1 - specificity' and positive predictive value versus negative predictive value are obtained by using different cut-offs for the log of the *P*-values obtained using each method; the results are shown in Figure 2. The sensitivity, specificity, positive predictive and negative predictive values for *SEPEA *analyses have better ranges than those for *GSEA *and *maxmean*. For a significant portion of the ranges of sensitivity and specificity for *GSEA *and *maxmean*, the *SEPEA *analyses provide higher sensitivity for a given level of false positives (a point on the '1 - specificity' axis). The same can be said about the portion of the ranges of the positive and negative predictive values of *maxmean *dominated by the *SEPEA *analyses. From the curves for *SEPEA_NT1 *and SEPEA_NT1*, we also observe the benefit of incorporating pathway network information. An updated Figure 2 that also includes results from *SEPEA_NT2 *and *SEPEA_NT3 *is provided as Figure S2 in Additional data file 3.

### Analysis using exposure of *Xenopus laevis *to cyclopamine data

Enriched KEGG pathways using *SEPEA_NT2 *and SEPEA_NT2* (which is essentially the *SEPEA_NT2 *analysis but does not make use of the network information of the pathways and is identical to the analysis of the Q2 test in [9]) methods were determined for a microarray dataset (see Materials and methods section) examining the consequences of inhibition of Sonic hedgehog (SHH) signaling by cyclopamine treatment of developing *Xenopus laevis *(Tables 5 and 6). Based on the specificity of cyclopamine to inhibit the SHH pathway, we expected to see the SHH signaling pathway significantly enriched; however, the *P*-value for this pathway was not significant using either method (*SEPEA_NT2 *and SEPEA_NT2*). This may be due to the time point at which gene expression was evaluated, which was optimized to evaluate downstream effectors of SHH pathway inhibition. Alternatively, this result may also reflect the limitation of the method when using only gene expression datasets, as several components of the SHH pathway, including Hedgehog (Hh) and Patched (PTCH), are known to be regulated at the protein level. Finally, when results obtained using *SEPEA_NT2 *versus SEPEA_NT2* are examined in the context of pathways linked to the SHH pathway (Figure S1 in Additional data file 2), we see that only the MAPK and Proteasome pathways are reachable from the SHH pathway by two and three edges, respectively, suggesting that results from *SEPEA_NT2 *may be more consistent with targets downstream of the SHH pathway. None of the other pathways listed in Tables 5 and 6 were reachable along the network of pathways (Figure S1 in Additional data file 2) from the SHH pathway. In fact, recent evidence suggests that SHH promotion of proliferation and differentiation in muscle [47] and gastric mucosal cells [48] is through transcription-independent activation of the MAPK/ERK pathway. This analysis suggests benefits of using pathway network information. Additional results from analysis of these data with *SEPEA_NT1*, *SEPEA_NT3*, *GSEA *and *maxmean *are provided in Additional data file 4.

**Table 5:** Enriched *X. laevis *pathways due to cyclopamine treatment using *SEPEA_NT2*

KEGG pathway ID	Pathway description	*P*-value
[path:xla03022]	Basal transcription factors	0.01
[path:xla04010]	MAPK signaling	0.02
[path:xla00460]	Cyanoamino acid metabolism	0.024
[path:xla00550]	Peptidoglycan biosynthesis	0.031
[path:xla02010]	ABC transporters	0.045
[path:xla03050]	Proteasome	0.05
[path:xla00982]	Drug metabolism - cytochrome P450	0.053
[path:xla00830]	Retinol metabolism	0.059
[path:xla04630]	Jak-STAT signaling	0.07
[path:xla04012]	ErbB signaling	0.1

Enriched KEGG [44] pathways (with *P*-value ≤ 0.1) due to cyclopamine treatment of developing *X. laevis*, designed to inhibit SHH signaling, using microarray data from GEO [45] [GEO:GSE8293]. *P*-values were obtained using the *SEPEA_NT2 *analysis with 1,000 randomizations to compute significance.

**Table 6:** Enriched *X. laevis *pathways due to cyclopamine treatment using SEPEA_NT2*

KEGG pathway ID	Pathway description	*P*-value
[path:xla00930]	Caprolactam degradation	0.006
[path:xla03030]	DNA replication	0.011
[path:xla00480]	Glutathione metabolism	0.016
[path:xla00561]	Glycerolipid metabolism	0.023
[path:xla03010]	Ribosome	0.045
[path:xla00982]	Drug metabolism - cytochrome P450	0.057
[path:xla00983]	Drug metabolism - other enzymes	0.057
[path:xla04012]	ErbB signaling	0.067
[path:xla03060]	Protein export	0.072
[path:xla00562]	Inositol phosphate metabolism	0.086
[path:xla04914]	Progesterone-mediated oocyte maturation	0.087
[path:xla04020]	Calcium signaling pathway	0.089

Enriched KEGG [44] pathways (with *P*-value ≤ 0.1) due to cyclopamine treatment of developing *X. laevis*, designed to inhibit SHH signaling, using microarray data from GEO [45] [GEO:GSE8293]. *P*-values were obtained using the SEPEA_NT2* analysis with 1,000 randomizations to compute significance.

### Analysis using OMIM breast cancer data

Genes associated with breast cancer were downloaded from the Online Inheritance in Man (OMIM) database [49]. This group of genes was pruned to include only those genes that participate in a pathway in the KEGG pathway database [44]. The list of genes used is provided in Table S5 in Additional data file 1. The *SEPEA *analysis was used to test whether there is an overabundance of 'important' (as defined by the scoring rules) breast cancer genes in pathways relative to the remaining set of genes that participate in some pathway in the KEGG pathway database [44]. Using these data, *SEPEA_NT3 *and SEPEA_NT3* (which is essentially the *SEPEA_NT3 *analysis but does not make use of the network information of the pathways and is very similar to those used in [7,9,11-22]) was used to find the enriched human pathways associated; the results are given in Table 7. Several of the pathways known to be important for breast cancer initiation and progression are significant using either method, such as the ErbB, p53, and apoptosis pathways. In contrast, the adherens junction, regulation of actin cytoskeleton, cell adhesion molecules, and focal adhesion pathways are significant using *SEPEA_NT3*, but are not considered significant using the SEPEA_NT3* method (*P *≤ 0.05). These pathways, in particular the focal and cell adhesion pathways, all deal with cell to cell communication and are thought to be key modulators of progression and invasion of malignant phenotypic characteristics [50]. In fact, several novel cancer chemotherapy drugs are being designed to specifically act on the focal adhesion pathway and many standard chemotherapy drugs modulate this pathway in conjunction with their primary mode of action [51]. So this analysis again suggests gains in the pathway enrichment analysis when network details of pathways are incorporated in the analysis.

**Table 7:** Enriched human pathways for susceptibility to breast cancer

KEGG pathway ID	Pathway description	SEPEA_NT3	SEPEA_NT3*
[path:hsa04370]	VEGF signaling pathway	1.69E-04	5.14E-04
[path:hsa04662]	B-cell receptor signaling	3.32E-04	3.51E-04
[path:hsa04630]	Jak-STAT signaling	8.91E-04	0.0417
[path:hsa04520]	Adherens junction	0.0014	0.1438
[path:hsa04810]	Regulation of actin cytoskeleton	0.0027	0.0717
[path:hsa04150]	mTOR signaling	0.0047	0.0052
[path:hsa04664]	Fc epsilon RI signaling	0.0081	5.99E-04
[path:hsa04510]	Focal adhesion	0.0103	0.0648
[path:hsa04012]	ErbB signaling	0.0103	8.51E-04
[path:hsa04210]	Apoptosis	0.0108	7.97E-04
[path:hsa03440]	Homologous recombination	0.0147	0.0016
[path:hsa04660]	T cell receptor signaling	0.0182	0.001
[path:hsa04010]	MAPK signaling	0.0183	0.0183
[path:hsa04910]	Insulin signaling	0.0191	0.0032
[path:hsa04514]	Cell adhesion molecules	0.0274	0.2407
[path:hsa04115]	P53 signaling	0.0306	0.0093
[path:hsa04620]	Toll-like receptor signaling pathway	0.0391	0.0193

Enriched KEGG [44] pathways (with *P*-value ≤ 0.05) obtained using genes from the OMIM database [49] that confer susceptibility to breast cancer. *P*-values were obtained using the *SEPEA_NT3 *and SEPEA_NT3* analysis.

## Conclusions

This paper presents a new method that uses biological data in order to find biochemical pathways that are relevant to the different responses of an organism to two different conditions. Biochemical pathways, instead of being treated as just sets of genes, are viewed as a network of interactions between proteins or metabolites. The extensive analysis using simulated and real data clearly demonstrates the utility of incorporating information on the interactions between the genes present in a pathway network.

## Materials and methods

### Notation

Assume there are *m *genes (identified by indices in the set *G *= {1, 2,..., *m*}) in the system and *n *array measurements (*n*_*c *_control and *n*_*t *_treated, *n*_*c *_+ *n*_*t *_= *n*) per gene. We will analyze one particular pathway made up of a subset *m*_*P *_of the *m *genes in the system. Without loss of generality, assume that these genes correspond to the first *m*_*P *_gene indices in *G*. The genes in this pathway are part of an underlying network of their gene products. On the basis of this network, gene *i *of the pathway is assigned a weight *w*_*i *_and a gene pair (*i *and *j*) is assigned two weights *d*_*ij *_(denoting a measure of the distance between these two genes on the network) and *e*_*ij *_(which is equal to 1 for a non-zero value of *d*_*ij*_). Each of the *m *genes is also assigned a value, *t*_*stat*, *k *_for gene *k *capturing the treatment effect on it as found in the observed data. This value obtained under the different null distributions (as defined in the next section) is denoted by *T*_*stat*, *i*_. The two scores, from the Heavy Ends Rule and the Distance Rule are denoted by *HER *and *DR*, respectively. They are a function of *t*_*stat*, *k*_. *HER*_*obs *_and *DR*_*obs *_denote those obtained from the observed experimental data while *HER*_*rand *_and *DR*_*rand *_those obtained from the different null distributions.

### Null hypotheses

Null hypotheses for the three statistical tests performed are given below and share similarities with those stated in [6].

Network test 1 (*NT1*): *T*_*stat*, *i*_, *i *= 1, 2,...*m *are identically distributed (and possibly dependent) with common distribution, *F*_*0 *_corresponding to the lack of association with the treatment, for each gene.

Network test 2 (*NT2*): *T*_*stat*, *i*_, *i *= 1, 2,...*m*_*p *_(only genes in the pathway) are identically distributed (and possibly dependent) with common distribution, *F*_*0 *_corresponding to the lack of association with the treatment, for each gene.

Network test 3 (*NT3*): *T*_*stat*, *i*_, *i *= 1, 2,...*m *are independent and identically distributed with a common distribution, *F *(which can take any form).

In all three hypotheses, *HER*_*obs *_and *DR*_*obs *_are each drawn from the distribution of *HER*_*rand *_and *DR*_*rand*_, respectively.

### Association value computations

For each gene we define by a pair of values (, ) corresponding to the association with the treatment in the context of the observed data. The association of any given gene with treatment is given in terms of the square of the two-sample t-statistic (similar to what has been done in [6,25,35]) and also shares similarities with the *maxmean *statistic defined in [10]. Mathematically:

(1)

(2)

(3)

where ,  are the sample mean gene expression for gene *g*_*i *_of the control and treated data, respectively, ,  are the associated standard deviations, *I*_*NT1 *_is equal to 1 when the *NT1 *test is being used and is equal to zero otherwise,  denotes the position of gene *i *in the sorted (in descending order) list of max(*t*_*stat*, *k*_, 0) over all the *m *genes, and, similarly,  denotes the position of gene *i *in the sorted (in ascending order) list of min(*t*_*stat*, *k*_, 0). *a *and *b *are parameters chosen empirically in order to control for the selection of the pathway with the most significant genes (relative to the other genes in the system). The first terms in the products on the right-hand side of Equation 2 will be called *importance *factors for a gene. These are values between 0 and 1. The functions 'mean' and 'var' refer to the standard definitions of mean and variance. The term *CF *denotes a (competitive) factor that is a measure of difference in the mean of differential expression of the genes in the pathway and that of the other genes in the system. Higher *CF *values indicate higher individual association values for genes in the pathway relative to the other genes and vice versa. Therefore, for similar values for changes in gene expression (*t*_*stat*, *i *_s) the power to detect treatment effect decreases as the *CF *factor decreases (or as more genes in the system are affected as a result of the treatment). For high values of the *CF *factor, parameter *a *controls the (decreasing) *importance *of genes along the sorted list. The parameter *b *provides a much steeper decrease in the *importance *of genes down the sorted list for small values of the *CF *factor.

Here, *t*_*stat*, *i *_is the standard two sample t-statistic. In some instances, the only information of the association of a gene with a treated condition may be just a summary statistic. For example, there are a set of known gene polymorphisms associated with breast cancer; in trying to identify pathways relevant for breast cancer, these genes would then be arbitrarily assigned a *t*_*stat*, *i *_equal to 1 while the other genes would be given values of 0. Note that in these situations, *n*, the number of array measurements per gene, is zero.

### Definition of the scoring rules

The score for linking the observed expression data to a given pathway has two components. The first component is called the Heavy Ends Rule score *HER*_*obs *_and will have a high value when a combination of the more 'important' genes (those associated with gene products close to a terminal of a pathway) is significantly associated with the treated condition. The second component called the Distance Rule score *DR*_*obs *_has a high value when the genes that are significantly associated with the treated condition have their gene products located close together. It is in fact the reciprocal of the weighted average distance between the genes in the network. The weights *w*_*i*_, *d*_*ij *_and *e*_*ij *_are defined in a subsequent section. Each score is defined as the maximum of individual expressions dependent either only on the genes whose expression increased due to the treatment or on the genes whose expression decreased as a result of the treatment. This should make it more robust to detect changes in both scale and location as discussed in [10]. The two scores are defined as:

(4)

For the *DR *score computation, 0/0 is defined to be equal to zero. The scores obtained under the null distributions are denoted by *HER*_*rand *_and *DR*_*rand *_and are defined as in Equation 4 with *t*_*i *_replaced by *T*_*i*_.

### Test statistic and significance evaluation

For each of the three hypotheses (*NT1*, *NT2 *or *NT3*) the test statistic is defined as:

(5)

where mean(*HER*) and std(*HER*) refer to the mean and standard deviation of the *HER *score for the given test and mean(*DR*) and std(*DR*) are those for the *DR *score.

For the *NT1 *and *NT2 *tests, multiple random samples of arrays are taken from the common set of treated and control data (without replacement) and randomly assigned to control or treated groups. For each random sample, the *T*_*stat*, *i*_s are calculated and then *HER*_*rand *_and *DR*_*rand *_are computed. The *NT1 *test requires *T*_*stat*, *i *_to be computed for all the *m *genes while the *NT2 *test requires computation for just the *m*_*P *_genes that are part of the pathway. For the *NT3 *test, multiple random samples of *m*_*P *_*T*_*stat*, *i *_s are drawn from the global set of *m *observed t_*stat*, *i*_.

The estimate of the *P*-value for each of the tests is computed as:

(6)

where *I*(*S*_*i *_≥ *S*_*obs*_) is an indicator function that equals 1 when the i^th ^randomly estimated test statistic value, *S*_*i*_, equals or exceeds the observed value and 0 otherwise. The estimation procedure used for the special case when the data are in the form of a list of differentially expressed genes or a list of genes associated with a disease is provided in Additional data file 1.

The way the significance computations are performed, tests *NT1 *and *NT3 *could be viewed as belonging to the class of 'competitive' hypotheses (as elaborated in the Background section) while *NT2 *could be viewed as a 'self-contained' hypothesis.

The method when applied to each of the three null hypotheses *NT1*, *NT2 *and *NT3 *is denoted by *SEPEA_NT1*, *SEPEA_NT2 *and *SEPEA_NT3*, respectively.

### Generation of simulated data

**Figure 3:**
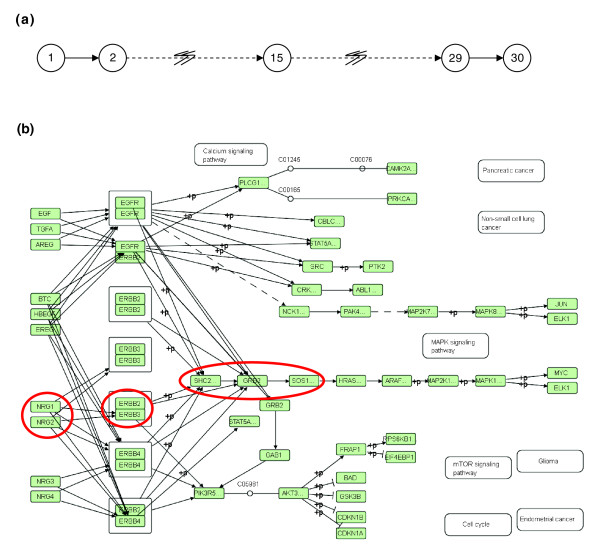
Schematic of networks used to generate simulated data. Illustrative schematic of the two pathways used to generate the simulated data. **(a) **The *Linear *network of 30 nodes/gene products, each of which is associated with one gene. The pair of squiggly lines across some arrows is used to indicate that there are more nodes that are not shown. **(b) **The Erbb signaling pathway from the KEGG pathway database [44]. The expressions of the genes associated with the nodes circled in red are correlated with each other and are the genes that were affected by the treatment.

Data were simulated from two genetic systems (*Linear *(*L*) and *ErbbSignaling *(*E*)) of 500 genes ( and ). Each system had two subnetworks of interest and each subnetwork was assumed to have no interactions with the other subnetwork. The *Linear *network had a set of 30 genes  that were connected in a linear fashion (Figure 3a). A set of 87 genes  in the *ErbbSignaling *network interacted in the same manner as described by the Erbb signaling pathway in the KEGG pathway database [44] (Figure 3b). Pathway enrichment analysis was performed on these two subnetworks.

Each set Λ and *H *had a subset of genes (with indices ), , whose expressions were perfectly correlated with each other (Σ^*L *^had *n*_*corr *_= 0 or 9 genes and Σ^*E *^had *n*_*corr *_= 7 genes). The gene expressions in the complement of each of the sets Σ^*L *^and Σ^*E*^, (Σ^*L*^)^*c *^and (Σ^*E*^)^*c*^, were assumed to be independent of each other even though some of them could be assumed to be known to have gene products that interact with gene products of genes in Σ^*L *^and Σ^*E*^. This could be justified by the fact that the interaction was not at the gene expression level and involved changes in the phosphorylation/binding states of the protein, for example. Let  denote the set of gene indices associated with the proteins circled in Figure 3b, ordered from left to right. The random variable defining the gene expression of gene *g*_*n *_is denoted by *X*_*n*_. Let N(*μ*, *σ*) represent the normal probability distribution with mean *μ *and standard deviation *σ*. Then data for all the 500 genes in each of the two systems were generated for one experiment under control conditions in the following manner:

(7)

Let *Φ* (*Φ*^*L *^and *Φ*^*E*^) denote the set of genes that are direct targets of the treatment. The total number of genes in the system affected by the treatment (that includes the set *Φ*) was chosen to be 50 and 10 for the *Linear *and *ErbbSignaling *networks, respectively. The effect of the treatment was to increase the mean of the expressions of the direct targets by a factor *pert*, *μ*' = *pert*·*μ*. Results from the assignment *pert *= 1.2 are discussed here while those resulting from other assignments are discussed in Table S3 in Additional data file 1. Let *U*^*L *^and *U*^*E *^denote a uniformly random selection of *n*_*corr *_genes from the sets *Λ* and *H*, respectively, let *V*_*n*_^*L *^and *V*_*n*_^*E *^denote sets of *n *genes drawn from the complements of the sets *Λ* and *H*, respectively, and let *Ø* denote the empty set. The details of the different correlation patterns considered here are given in Table 1. Patterns 1 and 3 were the correlation patterns that were favored by the scoring rules described in this paper.

All methods in this paper were coded using the Java programming language. For each combination of correlation pattern and *pert *assignment, 1,000 independent experiments were simulated. Each experiment involved the generation of *n*_*c *_= 5 control samples and *n*_*t *_= 5 treatment samples. For the randomization tests of each method, 1,000 randomizations were chosen. The performance measures chosen were the number of experiments out of the 1,000 performed that resulted in *P*-values for the test (Equation 6) below different chosen significance levels. The methods evaluated were *GSEA *[35], *maxmean *[10], *SEPEA_NT1*, *SEPEA_NT2 *and *SEPEA_NT3*. For the *SEPEA_NT1 *method, the parameters *a *and *b *in Equation 2 were empirically set to equal 2 and 5, respectively. Parameter *a *= 2 provides a quadratic decrease in the *importance *of genes along the sorted list for high values of the *CF *factor (when the mean changes in expression of the genes in the pathway are higher than that of the rest of the genes in the system). In the situation of low values of the *CF *factor, the value *b *= 5 was chosen such that the top 20% of genes in the sorted list approximately receive *importance *in the interval (0.2, 1) while the remaining genes receive weights in the interval (0, 0.2). Results from *GSEA *[35], *maxmean *[10] and *SEPEA_NT1 *are comparable because all test a similar null hypothesis. The main difference between these methods is that while *GSEA *and *maxmean *are blind to the structure of the biochemical pathway, *SEPEA-NT1 *is not.

### Assignment of network weights

The pathway network is represented by a set of nodes/gene products and set of edges between these nodes. The nodes represent gene products such as individual proteins or protein complexes. There is an edge from node/protein *u *to node/protein *v *if *u *transfers the signal it received immediately to *v *(either in the form of increasing the transcription of genes associated with *v*, changing the phosphorylation state of *v*, causing disassociation of *v *from a complex that it is part of) in the case of signaling pathways or that *u *and *v *catalyze two successive reactions in the case of metabolic pathways.

Let  denote the set of *P *nodes of the network and  denote the set of *N *genes associated with the nodes. The number of edges entering node *v*_*i *_is defined as its in-degree and the number of edges leaving *v*_*i *_is defined as its out-degree. We define a node to be a terminal node if either its in-degree or out-degree is zero.

Assume that each edge represents a unit distance between the two nodes that it connects. So if the shortest route between two nodes is via two edges in the pathway network, then the two nodes are said to be 2 units of distance apart. Note the phrase 'distance between a pair of nodes' is used to imply 'shortest distance between this pair', considering that there may be more than one path connecting the two nodes in the pathway network. Let *δ*_*j *_denote the shortest distance of node *v*_*j *_to a terminal node of the pathway. Let *G*(*v*_*i*_, *g*_*a*_) denote the indicator function, which is equal to 1 when gene *g*_*a *_is associated with node *v*_*i *_and is equal to 0 otherwise. The number of genes associated with node *v*_*i *_is denoted by *N*_*i*_. Let *s*_*ij *_denote the distance from node *v*_*i *_to node *v*_*j *_in the network. *s*_*ij *_is assigned a value of 0 either when *i *= *j *or when node *v*_*j *_is unreachable from node *v*_*i*_. Define the positive indicator function, *I*^+^(*x*), which is equal to 1 when *x *is positive and equal to 0 otherwise.

The weights for gene *g*_*a*_, *w*_*a*_, and gene pair (*g*_*a*_, *g*_*b*_), *d*_*ab *_and *e*_*ab*_, are given by:

(8)

The weight *w*_*a *_is defined such that genes associated with nodes closer to the terminal nodes have higher weights than those that are further away. The choice of a linear function to capture the intuition behind the *HER *is arbitrary and other functions will be experimented with as part of future work. The non-zero weights *d*_*ab *_for genes *a *and *b *are smaller if they are associated with gene products that are closer together in the pathway network than for pairs of genes whose gene products are further away.

### Statistical test for Distance Rule justification

Let the total number of pathways (nodes) in the network in Figure S1 in Additional data file 2 be denoted by *N*_*p*_. Denote the distance between pathways *i *and *j *on this pathway network by . Define  to be equal to zero if pathway *j *is not reachable from pathway *i*. Also define variable , which is equal to 1 for all non-zero values of the corresponding  and 0 otherwise. Perturbations to one pathway are transferred across the edges of the network to multiple pathways. Using human microarray data randomly chosen from the GEO database [45], we considered eight comparisons between two conditions (Table 1). For each comparison, the *DR *score was computed (Equation 4) for every human pathway on the network of pathways described above. In order to make the comparison possible across all the pathways, the *DR *scores obtained above using experimental data were normalized with *DR *scores obtained by setting the *T*_*stat*, *i *_values for all the genes equal to 1. Let the normalized *DR *score for pathway *i *be denoted by . A meta score can now be defined on the pathway network as follows:

(9)

Higher values of *meta_DR *would indicate that pathways with higher values of the normalized *DR *scores  are closer to each other. The significance of the obtained *meta_DR *scores are tested using random networks generated by the Markov-chain switching algorithm [52]. The properties of these random networks are that they have the same number of nodes and edges as the original pathway network and the degree sequence among all the nodes is also maintained. These networks differ, however, from the original network due to a number of random edge swaps across the network.

### GeneChip experiments

Cyclopamine powder (11-deoxyjervine; Toronto Research Chemicals Inc., North York, Ontario, Canada) was dissolved in 100% ethanol to a concentration of 5 mg/ml and this stock solution was stored at -20°C. A similar volume of 100% ethanol was stored at -20°C for use in vehicle control exposures. Approximately 200 tadpoles from each of two clutches (designated 'clutch A' and 'clutch B') of the species *Xenopus laevis *were obtained from Nasco Biology (Fort Atkinson, WI, USA) for a total of approximately 400 tadpoles. Animals were raised at an air temperature of 25 ± 1°C in tanks of 9 liters of tap water treated with Stress Coat (Aquarium Pharmaceuticals, Chalfont, PA, USA) and aged 1 day. Each day for three consecutive days, as animals reached stage 52 [53], the population of stage 52 individuals from each clutch was removed from the clutch tanks and divided in half indiscriminately, resulting in four exposure groups per day: a control group for clutch A; an experimental group for clutch A; a control group for clutch B; and an experimental group for clutch B. Each exposure tank had between 10 and 20 individuals, with 150 ml treated water per individual. After sorting into exposure tanks, 30 μl per animal of 5 mg/ml cyclopamine solution was added to all experimental tanks, and 30 μl per animal of 100% ethanol was added to each control tank. After 24 hours of exposure, animals were sacrificed by over-anesthesia with MS222, dried on a paper towel, then put into vials of RNAlater (Ambion, Austin, TX, USA). Vials were kept at 4°C overnight, then moved to -20°C for storage. Both hindlimb buds were dissected off each animal at the base of the limb using surgical scissors, placed in fresh vials of RNAlater, and returned to -20°C for continued storage. RNA extractions were performed using the RNeasy Mini Kit and optional RNase-Free DNase Set (QIAGEN, Valencia, CA, USA), with the following notes: limbs were put into a 1.5 ml microcentrifuge tube, residual RNAlater was pipetted off, and limbs were crushed with a homegenizer in 200 μl buffer RLT, then 300 μl more buffer RLT was added; and elution was carried out with two washes of 50 μl RNase-free water. Extracted total RNA was stored at -80°C and transferred to the WM Keck Foundation Biotechnology Resource Center, Affymetrix Resource Center (Yale University, New Haven, CT), where they were again run through DNase treatment. Four control-experimental pairs of samples were chosen, from a total of 12 pairs, based on quantity and quality of RNA as determined by analysis on an Agilent 2100 Bioanalyzer RNA Nano chip (Agilent Technologies Inc., Santa Clara, CA, USA). Samples in each pairwise comparison were extracted from the same number of limbs, were from the same clutch, were exposed to cyclopamine solution or ethanol on the same day, and their total RNA was extracted in the same batch of extractions. The eight chosen samples were each hybridized to an Affymetrix^® ^GeneChip^® ^*Xenopus laevis *Genome Array (Affymetrix, Santa Clara, CA, USA) using 3 μg total RNA. Data have been deposited in the National Center for Biotechnology Information, NCBI GEO with series record ID [GEO:GSE8293].

## Abbreviations

*DR*: Distance Rule; GEO: Gene Expression Omnibus; *GSEA*: gene set enrichment analysis; *HER*: Heavy Ends Rule; KEGG: Kyoto Encyclopedia of Genes and Genomes; MAPK: mitogen-activated protein kinase; *NT*: network test; OMIM: Online Mendelian Inheritance in Man; *SEPEA*: structurally enhanced pathway enrichment analysis; SHH: Sonic hedgehog.

## Authors' contributions

RT and JMG designed and evaluated the research with important suggestions from CJP and FMP. RT implemented the research and drafted the manuscript. GFS performed the *Xenopus laevis *experiments. All the authors read and approved the final manuscript.

## Additional data files

The following additional data are available with the online version of this paper: a Word document that provides a section on a particular estimation of *P*-values and additional tables of results (Additional data file 1); a figure of the network of pathways in the KEGG pathway database [44] (Additional data file 2); a figure that demonstrates the receiver-operator characteristics of the different methodologies used (Additional data file 3); a table with the *P*-values for KEGG [44] pathways after cyclopamine treatment of developing *X. laevis*, designed to inhibit SHH signaling, using microarray data from GEO [45] [GEO:GSE8293] (Additional data file 4).

## Supplementary Material

Additional data file 1 Estimation of *P*-values and additional tables of results. Click here for file

Additional data file 2 The nodes of this network are pathways while the edges indicate the transfer of signal or material between the pathways. Click here for file

Additional data file 3 **(a) **Sensitivity versus '1 - specificity' of enriched pathways that are predictive of survival from lung cancer for the six methods: *SEPEA_NT1*, SEPEA_NT1*, *SEPEA_NT2*, *SEPEA_NT3*, *GSEA *and *maxmean*. SEPEA_NT1* is the same analysis as in *SEPEA_NT1 *except that the pathway network information was not used. **(b) **Positive predictive power (ppp) versus negative predictive power (npp) for the same data and using the same methods of analysis as in (a). Click here for file

Additional data file 4 *P*-values for KEGG [44] pathways after cyclopamine treatment of developing *X. laevis*, designed to inhibit SHH signaling, using microarray data from GEO [45] [GEO:GSE8293]. *P*-values were obtained using *SEPEA_NT1*, *SEPEA_NT2*, *SEPEA_NT3*, *GSEA *and *maxmean *analyses with 1,000 randomizations to compute significance. Click here for file
