# Supplementary material for: Choosing the right path: enhancement of biologically relevant sets of genes or proteins using pathway structure
Source: Genome Biol. 2009 Apr 24;10(4):R44. doi: 10.1186/gb-2009-10-4-r44 (PMC2688935; doi:10.1186/gb-2009-10-4-r44)
Supplement: Additional data file 1 — Estimation of P-values and additional tables of results. [file gb-2009-10-4-r44-S1.doc]

**Estimation of p-value for the *SEPEA_NT3* method using significance list data**

This computation scheme assumes that the data is in the form of a list of differentially expressed genes or a list of genes associated with a disease (of size *n_d_*). Let there be *m* genes in the system under consideration (e.g. the set of all genes involved in some biochemical pathway on the KEGG pathway database). Assume that the pathway under consideration has *m_p_* genes associated with it. Let $S$ be the random variable denoting the score, which follows the null hypothesis distribution and let *s* be the observed score. Let $C$ be the random variable denoting the number of the common genes of a random set of size *n_d_* with the pathway under consideration.

Then,

$$\text{p}-\text{value}=\Pr\{S\geq s\}=\sum_{c=0}^{\min\{m_{p},n_{d}\}} \Pr\left\{\frac{S\geq s}{C=c}\right\}\Pr\{C=c\}$$

$$\Pr\{C=c\}=\frac{\left(\begin{matrix} m_{p} \\ c \end{matrix}\right)\left(\begin{matrix} m-m_{p} \\ n_{d}-c \end{matrix}\right)}{\left(\begin{matrix} n \\ n_{d} \end{matrix}\right)}$$

The terms in the formula for $\Pr\{C=c\}$ are the standard binomial coefficients. Each of the terms $\Pr\left\{\frac{S\geq s}{C=c}\right\}$ is estimated using 1000 simulations. For each of these terms, during one of the 1000 simulations, *c* genes are randomly picked from the genes involved in the given pathway network.

**Table S1**: Different correlation patterns (6-8) considered for the generation of simulated data along with the underlying networks, the set of correlated genes, *Σ* and the set of genes that are the targets of the treatment, *Φ* . *U^L^* denotes a uniformly randomly drawn set of 9 genes drawn from the set of genes associated with the pathway displayed in Figure 4a. *V_41_^L^* denotes a set of 41 randomly drawn genes from the set of 470 genes not associated with the pathway displayed in Figure 4a.

| Pattern no. | Network | Correlated set (*Σ*) | Target set (*Φ*) |
| --- | --- | --- | --- |
| 6. | *Linear* | {*g_1_*,*g_12_*,*g_13_*,…,*g_17_*,*g_18_,* *g_30_*} | $\{g_{1}\}\cup V_{41}^{L}$ |
| 7. | *Linear* | {*g_11_*,…,*g_19_*} | $\{g_{11}\}\cup V_{41}^{L}$ |
| 8. | *Linear* | {*g_5_*,*g_7_*,…,*g_19_*, *g_21_*} | $\{g_{5}\}\cup V_{41}^{L}$ |

**Table S2**: Power estimates for the *SEPEA_NT1*, *GSEA*, *SEPEA_NT2* and *Maxmean* (only for pattern 5) methods (in terms of the number of experiments out of a 1000 that gave p-values for the randomization tests below a nominal size, *α* = 0.01 and 0.05 level). The estimates for *Maxmean* (for patterns 6-8) are given at an empirical size of 0.07 (nominal size of 0.001) and for *SEPEA_NT3* are given at an empirical size of 0.05 (nominal size of 0.001). These are results from the simulation in which the treatment resulted in an over-expression of the mean expression of the target genes by a factor, *pert*=1.2. The methods were evaluated on correlation patterns 5-8.

| Pattern no. | *α* | *SEPEA_NT1* | *GSEA* | *Maxmean* | *SEPEA_NT2* | *SEPEA_NT3* |
| --- | --- | --- | --- | --- | --- | --- |
| 5 | 0.01 | 528 | 520 | 391 | 643 | 200 |
|  | 0.05 | 931 | 952 | 857 | 969 | 395 |
| 6 | 0.01 | 225 | 182 | 29 | 257 | 9 |
|  | 0.05 | 470 | 529 |  | 565 |  |
| 7 | 0.01 | 193 | 196 | 45 | 261 | 15 |
|  | 0.05 | 457 | 528 |  | 607 |  |
| 8 | 0.01 | 121 | 173 | 38 | 141 | 5 |
|  | 0.05 | 249 | 523 |  | 291 |  |

**Table S3**: Variation of power estimates for the *SEPEA_NT1*, *GSEA* and *SEPEA_NT2* methods (in terms of the number of experiments out of a 1000 that gave p-values for the randomization tests below a nominal size *α* = 0.01 and 0.05 level), *Maxmean* at an empirical size of 0.07 (nominal size of 0.001) and *SEPEA_NT3* at an empirical size of 0.05 (nominal size of 0.001)with size of the effect of the treatment, *pert* on the mean of the expressions of the target genes. All results are for the correlation pattern 1.

| Pert level | *Α* | *SEPEA_NT1* | *GSEA* | *Maxmean* | *SEPEA_NT2* | *SEPEA_NT3* |
| --- | --- | --- | --- | --- | --- | --- |
| 1.1 | 0.01 | 82 | 55 | 142 | 83 | 185 |
|  | 0.05 | 230 | 207 |  | 251 |  |
| 1.2 | 0.01 | 328 | 188 | 52 | 357 | 321 |
|  | 0.05 | 610 | 510 |  | 686 |  |
| 1.5 | 0.01 | 886 | 697 | 48 | 880 | 328 |
|  | 0.05 | 1000 | 995 |  | 1000 |  |
| 2 | 0.01 | 943 | 804 | 18 | 912 | 237 |
|  | 0.05 | 1000 | 1000 |  | 1000 |  |
| 3 | 0.01 | 920 | 782 | 18 | 911 | 179 |
|  | 0.05 | 1000 | 1000 |  | 1000 |  |

**Table S4**: Variation of power estimates for *SEPEA_NT1*, *GSEA* and *SEPEA_NT2* methods (in terms of the number of experiments out of a 1000 that gave p-values for the randomization tests below a nominal size *α* = 0.01 and 0.05 level), *Maxmean* at an empirical size of 0.07 (nominal size of 0.001) and *SEPEA_NT3* at an empirical size of 0.05 (nominal size of 0.001) with signal to noise μ/σ of the expressions of all the genes in the network. These are results from the simulation in which the treatment resulted in an over-expression of the mean expression of the target genes by a factor, *pert*=1.2. All results are for the correlation pattern 1.

| *μ/σ* | *Α* | *SEPEA_NT1* | *GSEA* | *Maxmean* | *SEPEA_NT2* | *SEPEA_NT3* |
| --- | --- | --- | --- | --- | --- | --- |
| 10 | 0.01 | 328 | 188 | 52 | 357 | 321 |
|  | 0.05 | 610 | 510 |  | 686 |  |
| 4 | 0.01 | 53 | 34 | 121 | 52 | 163 |
|  | 0.05 | 161 | 153 |  | 176 |  |
| 2 | 0.01 | 18 | 18 | 70 | 16 | 118 |
|  | 0.05 | 89 | 73 |  | 88 |  |

**Table S5**:

OMIM [48] breast cancer genes used in the analysis in the paper

| **NCBI-ID** | **Gene Name** |
| --- | --- |
| 207 | AKT1 |
| 672 | BRCA1 |
| 675 | FANCD1 |
| 999 | CDH1 |
| 3845 | KRAS2 |
| 5290 | PIK3CA |
| 7157 | TP53 |
| 8438 | RAD54L |
| 8493 | PPM1D |
