# Supplementary figures and images for: Choosing the right path: enhancement of biologically relevant sets of genes or proteins using pathway structure
Source: Genome Biol. 2009 Apr 24;10(4):R44. doi: 10.1186/gb-2009-10-4-r44 (PMC2688935; doi:10.1186/gb-2009-10-4-r44)

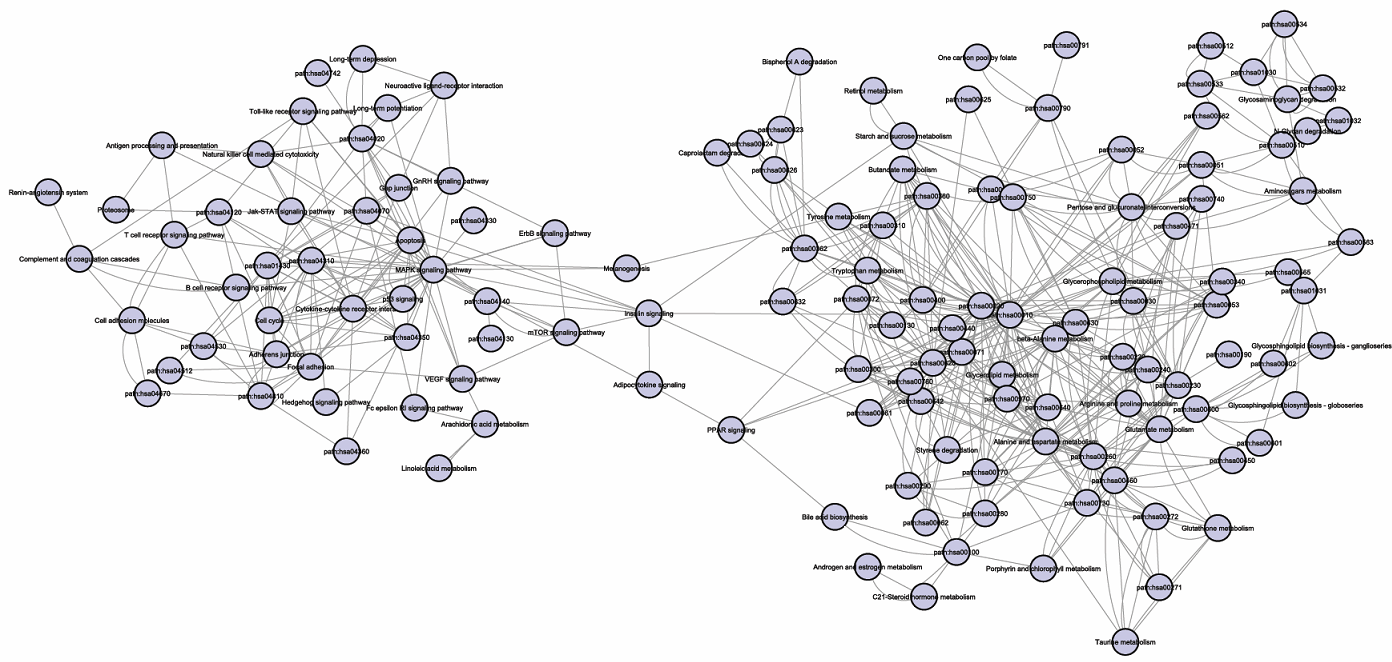

Supplement: Additional data file 2 — The nodes of this network are pathways while the edges indicate the transfer of signal or material between the pathways. [file gb-2009-10-4-r44-S2.png]
